# Making the Auroras glow: regulation of Aurora A and B kinase function by interacting proteins

**DOI:** 10.1016/j.ceb.2009.09.008

**Published:** 2009-12

**Authors:** Mar Carmena, Sandrine Ruchaud, William C Earnshaw

**Affiliations:** Wellcome Trust Centre for Cell Biology, University of Edinburgh, Michael Swann Building, King's Buildings, Mayfield Road, Edinburgh EH9 3JR, Scotland, UK

## Abstract

The conserved Aurora family of protein kinases have emerged as crucial regulators of mitosis and cytokinesis. Despite their high degree of homology, Aurora A and B have very distinctive localisations and functions: Aurora A associates with the spindle poles to regulate entry into mitosis, centrosome maturation and spindle assembly; Aurora B is a member of the Chromosomal Passenger Complex (CPC) that transfers from the inner centromere in early mitosis to the spindle midzone, equatorial cortex and midbody in late mitosis and cytokinesis. Aurora B functions include regulation of chromosome–microtubule interactions, cohesion, spindle stability and cytokinesis. This review will focus on how interacting proteins make this functional diversity possible by targeting the kinases to different subcellular locations and regulating their activity.

## Introduction

Successful cell division depends upon the function of key regulatory protein kinases. The best known of these, Cyclin dependent kinases (CDKs), Polo-like kinases (PLKs) and Aurora kinases control mitotic entry and ensure the accurate coordination of chromosomal and cytoskeletal events, leading to the correct partition of the genetic material into two daughter cells. Defects in the function and expression of these kinases result in aneuploidy and have been linked to tumorigenesis [1], making them attractive targets for the development of new anti-cancer treatments [2]. The study of their functions and regulation is revealing a network of interactions between the pathways controlled by each kinase. Many recent studies of these kinases have focused on the development and characterisation of Aurora-specific and Aurora-selective inhibitors. These have been recently reviewed [3] and will not be covered here.

The Aurora family of Ser/Thr kinases has emerged as crucial regulators of essential processes ranging from mitotic entry to cytokinesis. The number of family members varies depending on the species: fungi have one Aurora gene, whereas in most higher eukaryotes the family has branched, with Auroras A and B adopting different subcellular localisations and functions (see below). In mammals, a third member — Aurora C — that most closely resembles Aurora B, is normally expressed primarily in testis.

**Figure 1:**
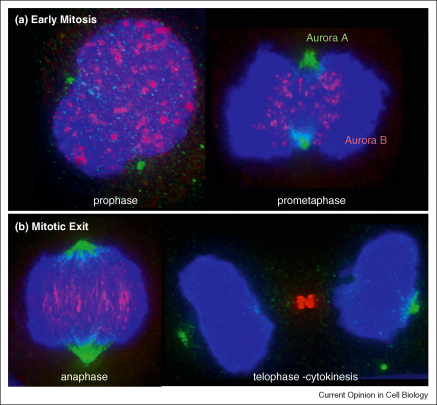
Distribution of Aurora A and Aurora B in mitotic HeLa cells.

**Figure 2:**
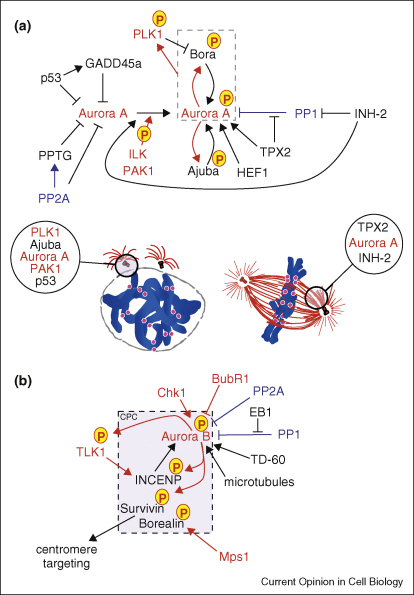
The major regulators of **(A)** Aurora A and **(B)** Aurora B kinases. Protein kinases are indicated in red and phosphorylation events by red arrows. Protein phosphatases are indicated in blue.

Aurora A and B are very similar proteins in sequence and structure, sharing 70% identity in the catalytic domain. However despite their similarities they have quite distinct localisations and functions during mitosis (Figure 1). Aurora A associates with the spindle poles and functions in mitotic entry, centrosome maturation and separation and spindle bipolarity [4,5]. Aurora B is the enzymatically active member of the Chromosomal Passenger Complex (CPC), which includes the scaffolding protein INCENP and the targeting subunits Survivin and Borealin/Dasra B. The CPC associates with the inner centromere until metaphase and then transfers to the spindle midzone, equatorial cell cortex and midbody in late mitosis and cytokinesis [5,6]. Aurora B functions include regulation of chromosome interactions with microtubules, chromatid cohesion, spindle stability and cytokinesis [6]. How such similar proteins can occupy these diverse functional cellular niches is partly explained by their association with specific cofactors (Figure 2) that act as targeting and activating subunits (see below).

Surprisingly, most of these cofactors associate with highly conserved residues in the Aurora catalytic domain rather than the more variable N-terminus. Structural mutagenesis analysis revealed that a single amino acid difference (G198 in human Aurora A/N142 in human Aurora B) is responsible for the differences in basal kinase activity [7] and allows TPX2 to discriminate between Aurora A and B [7,8]. An Aurora A G198N mutant shows classical Aurora B localisation, partially rescues Aurora B loss of function and associates with the CPC subunits INCENP and Survivin [9,10]. Interestingly, despite showing several ‘Aurora B-like’ features, the yeast enzymes have a Glycine in the equivalent residue, like Aurora A [7].

Indeed, Aurora A and B normally exhibit limited functional interchangeability. Aurora A can phosphorylate some Aurora B substrates *in vitro* (i.e. Histone 3, CENP-A, INCENP, Survivin) and both kinases act upon common substrates at different times during mitosis (i.e. MCAK, Kif2, RASSF1A). This partial overlap in substrate specificity could have important implications when the kinases are misexpressed or mislocalised. Nonetheless, the two kinases are fundamentally distinct, and Aurora A, but not Aurora B can function as a classical oncogene when overexpressed [11].

Regulation of Aurora kinases occurs at the levels of gene expression, targeting, local activation and degradation. Degradation of Aurora kinases depends mainly on the Anaphase Promoting Complex/Cyclosome (APC/C) with its auxiliary subunit Cdh1 [12–15]. It has been claimed that degradation of Aurora A is the main contribution of Cdh1 to the regulation of mitotic exit [16^••^]. When stabilised by depletion of Cdh1 Aurora A accumulates on the spindle poles and microtubule asters persist until G1. In these cells there is an increased accumulation of Aurora B in the equatorial cortex [16^••^]. Removal of Aurora B from the chromosome arms in early mitosis and subsequent transfer to the central spindle involve a distinct CUL3-containing SCF ubiquitin ligase [17].

Degradation of Aurora A is regulated by phosphorylation in two different ways. Firstly, its degradation requires dephosphorylation of a serine residue in the A-Box (Ser53 in *Xenopus*, Ser 51 in human Aurora A), probably by PP2A [18^•^]. An alternative pathway for Aurora A degradation involves phosphorylation of AIP1 by GSK3beta [19]. AIP1 localises at the centrosome where it binds and downregulates Aurora A early in mitosis [20].

Full activation of both Auroras requires binding to specific protein cofactors. These frequently induce a conformational change in the kinase domain, leading to auto-phosphorylation of a Threonine residue in the T-loop (T288 in human Aurora A, T232 in Aurora B). Most importantly, kinase activation at specific cellular locations during mitosis requires timely association with interacting proteins. In the following sections we will focus on these interactors and the way in which they modulate the function of Aurora kinases.

## Aurora A functions

Aurora A was discovered in a screen for *Drosophila* mutations affecting the poles of the mitotic spindle (hence the name referring to the Aurora Borealis [21]), and many Aurora A functions are related to its ability to bind microtubules coupled with its location at spindle poles. Aurora A microtubule binding and centrosomal targeting depends on numerous auxiliary proteins (see next section).

### Mitotic entry

Aurora A phosphorylation of CDC25B on Ser353, promotes the activation of this critical phosphatase [22,23], leading to the activation of centrosome-associated CyclinB–CDK1 [24]. Activation of PLK1 by Aurora A in complex with its auxiliary cofactor Bora in G2 also contributes to the final activation of CyclinB–CDK1 [25^••^,26^••^] as PLK1 promotes degradation of the CDK1 inhibitor Wee1 [27].

### Centrosome maturation

Centrosomes increase both in size and in microtubule-nucleating capacity just before mitotic entry [28]. Aurora A contributes to this by recruiting pericentriolar material (PCM) proteins including Centrosomin [29], LATS2, TACC and NDEL1 [30–32]. The Ser/Thr kinase LATS2 is required for recruitment of gamma-tubulin, a key step in increasing the microtubule-nucleating capacity of the centrosome. NDEL1 is required for the recruitment of the microtubule-severing protein katanin p60, promoting microtubule remodelling. Interestingly, NDEL1 also contributes to the centrosomal targeting of TACC [32], which, when phosphorylated, forms a complex with XMAP215/Msps and promotes microtubule growth.

### Bipolar spindle formation

Centrosome separation and bipolar spindle formation require both sliding forces between anti-parallel microtubules and cortical forces that act on the asters. Integrity of the astral microtubules connecting the centrosomes with the cell cortex depends on Aurora A [33]. The bipolar kinesin Eg5 can slide anti-parallel microtubules and is involved in centrosome separation [34]. Eg5 is an Aurora A substrate, however, there is no direct evidence for a function of this phosphorylation in centrosome separation.

### Chromosomal pathway of spindle formation

This pathway, which is particularly important in cells lacking centrosomes, depends on a Ran-GTP gradient that locally activates MAPs involved in bipolar spindle assembly. In *Xenopus* extracts, Aurora A drives this Ran-GTP-dependent bipolar spindle formation by a mechanism involving gamma-tubulin and TPX2 [35]. Aurora A promotes the formation and function of a complex containing TPX2, Eg5, HURP (a microtubule bundling factor) and XMAP215 (a microtubule stabiliser) that is essential for spindle bipolarity [36]. Aurora A phosphorylation blocks binding of the HURP C-terminus to its own N-terminus, thereby allowing its interaction with microtubules [37]. Aurora A phosphorylation of the microtubule-destabilising kinesin 13 MCAK promotes its localisation to spindle poles [38] and also controls the stability of Aster Associated Protein (ASAP) [39]. Both MCAK and ASAP are required for bipolar spindle assembly [38,39].

## Regulation by Aurora A interacting proteins

The best studied protein cofactor for Aurora A is TPX2, a MAP that targets the kinase to the mitotic spindle (but not the centrosome) and activates it [40,41]. TPX2 has a dual role in Aurora A activation. Its N-terminus binds the kinase, inducing a conformational change that facilitates auto-phosphorylation of Thr288 in the T-loop [42,43]. Bound TPX2 then shields this residue from dephosphorylation by PP1 on entry into mitosis [42,43].

Ajuba is a multifunctional protein that interacts with the N-terminus of Aurora A at the centrosome. Ajuba phosphorylation by Aurora A promotes kinase auto-phosphorylation and its full activation [24]. Ajuba is a MAP that tracks on assembling MTs from the centrosome to the kinetochore, where it partially colocalises with Aurora B and BubRI [44]. Although Ajuba binds these proteins *in vitro* it is unclear whether this binding has a role at centromeres *in vivo*.

Bora was originally identified due to its role in *Drosophila* asymmetric cell division [45]. Bora binding and phosphorylation by Aurora A is also required for full kinase activation. In addition, both Bora and Aurora A are required for PLK1 activation at the centrosome in G2 [25^••^]. Bora binding to PLK1 controls Aurora A access to the PLK1 T-loop, where Aurora A phosphorylates Thr210, leading to full PLK1 activation [26^••^]. Interestingly, Bora degradation appears to involve a negative feedback loop: PLK1 phosphorylation of Bora creates a recognition site for the E3 ubiquitin ligase SCF-betaTrCP [46].

Inhibitor 2 is a PP1 regulatory subunit that binds and activates Aurora A *in vitro* [47]. During mitosis it localises to the mitotic spindle, midzone and midbody, where it has been suggested to balance the activities of PP1 and Aurora B [48].

Curiously, three proteins with previously well-defined roles at focal adhesions also colocalise with, and appear to regulate, Aurora A at the centrosome and/or microtubule asters. HEF-1 binding and activation of Aurora-A is required for phosphorylation and activation of HDAC6, a tubulin deacetylase that promotes ciliary disassembly at the basal body [49^•^]. This is the only non-mitotic function of Aurora A thus far described in vertebrates.

The other two focal adhesion components that regulate Aurora A are both protein kinases. Inhibition or depletion of ILK (Integrin-like kinase) causes mitotic spindle defects by disrupting interactions between Aurora A and TACC3/ch-TOG [50]. PAK1 is a member of the PAK–PIX–GIT complex that targets and regulates focal adhesions. This complex is also required for centrosome maturation [51]. PAK1 becomes activated at the centrosome and promotes activation of Aurora A by phosphorylation on Thr288 and Ser342 [51].

Not all Aurora A interacting factors activate the kinase. Two protein phosphatases bind and inhibit Aurora A. PP1 dephosphorylation of T288 keeps Aurora A inactive in interphase. At NEB TPX2 alleviates this inhibition by binding to Aurora A and blocking PP1 access [42,43]. PP2A can bind Aurora A *in vivo* and the two are mutually dependent for their centrosomal localisation [18]. PP2A promotes Aurora A inactivation by dephosphorylating the kinase directly [42] and also by stabilising PTTG1 (pituitary tumor transforming gene 1), a mammalian securin protein that inhibits Aurora A *in vivo* and *in vitro* [52^•^].

Amongst its multitudinous other activities, p53 regulates transcription of GADD45a, a protein that binds and strongly inhibits Aurora A [53]. p53 also colocalises with Aurora A at the centrosome, where it may inhibit the kinase directly. TPX2 binding shields the Aurora A T-loop from p53 inhibition [54]. This could explain why the transforming capability of Aurora A in human cells is detected mainly when the p53 pathway is compromised.

## Regulation of Aurora B by interacting proteins

### The core CPC

Aurora B localisation and activation requires the three regulatory subunits of the CPC: INCENP, Survivin and Borealin/Dasra B [55–61]. INCENP, Borealin and Survivin form a 1:1:1 complex through a three-helix bundle involving the N-termini of INCENP and Borealin, and the C-terminus of Survivin [62^••^]. This core subcomplex is stable and can target to centromeres *in vivo* (Z Xu *et al.*, unpublished data), and association with Aurora B occurs via contacts with the INCENP IN-Box [57].

INCENP binding to Aurora B increases basal activation of the kinase, which then achieves full activity via a feedback loop following phosphorylation of the bound INCENP at a TSS motif proximal to the C-terminus [63,64]. Borealin/Dasra B is suggested to promote local clustering that leads to Aurora B auto-activation at the centromere [64,65]. Phosphorylation of Borealin by the checkpoint kinase Mps1 leads to increased activation of the kinase at the centromere [66^•^] by an as yet unknown mechanism. Survivin appears to be involved in targeting the CPC to centromeres [67], however its role, if any, in regulating Aurora B activity remains controversial [68–71]. There is a vast literature examining the roles of Survivin in apoptosis regulation, inspired largely by its having a BIR domain resembling that found in other IAP (inhibitor of apoptosis) proteins. However, analysis of Survivin knockout mice, which are early-embryonic lethals [58] and detailed analysis of a conditional knockout of Survivin in DT40 cells [71] have failed to confirm any role for this protein in the regulation of apoptosis. As one possible solution to this paradox, it has been suggested that Survivin, when present in the cytoplasm as a dimer, may exert anti-apoptotic functions under certain circumstances, whereas when present as a monomer in the CPC, its anti-apoptotic functions (if any) may be abrogated [72].

The recent discovery of CPC paralogues has posed a conundrum that remains to be solved. As stated above, mammals, which have single genes for INCENP, Borealin and Survivin, have a third Aurora kinase, Aurora C, whose only known activities in non-transformed cells are found in testis [73–77]. Immunostaining reveals that this kinase exhibits a CPC-like distribution similar to Aurora B during meiosis [77], and Aurora C knockout mice were viable, though sterile [76]. Paradoxically, a number of model organisms, including *Drosophila*, *Xenopus*, chicken and zebra fish, have paralogues of other CPC components, including Survivin, Borealin and INCENP, but only the canonical Aurora A and Aurora B. These have been little studied, but the Borealin paralogues Dasra A and Australin appear, like Aurora C to be meiosis-specific [61,78]. Survivin2 has been reported to have a role in haematopoiesis [79], but is not expressed in chicken DT40 cells (J. Bergmann, K. Samejima and WCE, unpublished results). *INCENP2* has thus far only been described in chicken, where it is expressed in somatic cells and is unable to substitute for the canonical INCENP (Z Xu *et al.*, unpublished data).

### Other Aurora B regulators

Two kinases are involved directly in the activation of Aurora B. *C. elegans* Tousled-like kinase (TLK-1) is a substrate activator that increases Aurora B activity in an INCENP-dependent manner [80]. The checkpoint kinase Chk1 phosphorylates Aurora B and increases its activity at the centromere [81]. The mechanisms by which these kinases increase Aurora B activity remain to be determined.

The little-studied fifth chromosomal passenger TD-60 (telophase disc-60 kD) was first discovered as the target of a human auto-antibody [82]. When TD-60 was cloned, its cDNA was shown to encode a sequence that could be modelled as an RCC1-like GTPase exchange factor (GEF) [83]. Depletion of the protein by RNAi led to a penetrant prometaphase mitotic arrest. A more recent study has shown that full activation of Aurora B kinase *in vitro* requires both TD-60 and microtubules [84^••^]. In this assay TD-60 apparently did not function as a GEF, and the significance of its structural similarity to RCC1 remains uncertain. TD-60 binds INCENP and is also required for the centromeric localisation of the CPC and Haspin kinase. The involvement of microtubules in Aurora B activation was further supported by studies *in vivo* in *Xenopus* S3 cells during anaphase using a proximity-ligation *in situ* assay [85^••^].

The protein phosphatases PP1 and PP2A bind and inhibit Aurora B [86,87]. In anaphase, binding of the microtubule plus-end-binding protein EB1 shields the kinase T-loop from PP2A dephosphorylation [87]. The checkpoint protein BubR1 inhibits Aurora B activity (as detected by CENP-A phosphorylation) at the kinetochore. This may promote the formation of stable microtubule–kinetochore attachments (see below) [88].

## Aurora B functions

### Spindle assembly

Aurora B and the CPC are required for stability of the bipolar mitotic spindle [60,89]. In *X. laevis* egg extracts, Aurora B phosphorylates and inhibits two proteins involved in the chromatin-driven spindle assembly pathway, the microtubule-destabilising protein Stathmin/Op18 and the kinesin-13 microtubule depolymerase MCAK [90,91]. However, bipolar spindles can form when CPC components are depleted (Z Xu *et al.*, unpublished data) [60,71,89]. The role of the CPC in maintaining bipolar spindle stability remains unknown.

### Promoting chromosome bi-orientation by correcting mis-attachments

One of the best-described functions of Aurora B is in promoting chromosome bi-orientation. This was first proposed as a result of studies in budding yeast suggesting that Aurora activity was required for kinetochores to release bound microtubules [92]. Those authors suggested a physical model in which Aurora B in the centromere would continually promote disruption of kinetochore–microtubule attachments until the bi-oriented chromosome came under tension, stretching the kinetochores away from the inner centromere and removing them from the ‘zone of influence’ of the Aurora B. This was proposed to lead to stabilisation of the attachments. Two subsequent studies provided evidence consistent with this hypothesis by looking at the distribution of the microtubule depolymerising kinesin-13 MCAK phosphorylated by Aurora B on kinetochores that were or were not under tension [93,94].

This very prescient model has recently been confirmed in a particularly elegant manner by tethering Aurora B at differing locations within the centromere [95^••^]. If tethered in the inner centromere, Aurora B is active, but does not efficiently promote detachment of microtubules from the kinetochore. In contrast, if tethered within the kinetochore itself, the kinase efficiently promotes microtubule detachment, thereby interfering with stable bi-orientation of the chromosomes on the spindle. These observations provided clear support for the hypothesis that Aurora B activity can be regulated by adjusting its physical separation from its substrates [95^••^].

How does Aurora B promote microtubule release by kinetochores? The formation of incorrect attachments of vertebrate chromosomes to the spindle appears to be relatively common in early mitosis, and cells employ redundant mechanisms to correct these errors. Aurora B phosphorylation of the N-terminal region of Hec1/Ndc80 decreases its affinity for microtubules [96,97]. Since Hec1/Ndc80 is a key microtubule-binding component of the KMN network [97], this is predicted to promote microtubule release. Aurora B also promotes the correction of microtubule mis-attachments by regulating the activity of MCAK [93,94,98,99] and by regulating the recruitment of Kif2b to outer kinetochores [100].

### Spindle assembly checkpoint

The spindle assembly checkpoint (SAC) is a surveillance mechanism that delays anaphase onset until all chromosomes are bi-oriented and under tension (for a comprehensive review of the SAC signalling and the possible roles of Aurora B see the review by Nezi and Musacchio in this issue [101]). There has been an energetic argument over the years regarding whether the SAC senses only kinetochore occupancy with microtubules or can also sense decreased tension at microtubule–kinetochore attachments. Part of the reason for the complexity of this argument is that tension regulates the stability of microtubule attachments (see above), and therefore treatments that lower spindle tension would also favour sporadic detachment of microtubules from bi-oriented chromosomes.

The budding yeast Aurora kinase, Ipl1 is required for SAC function in response to a lack of tension [102,103]. In vertebrates, the use of small molecule inhibitors or inhibitory antibodies revealed a similar requirement for Aurora B in the checkpoint response to microtubule stabilising agents that induce a lack of tension [104–106]. Thus, Aurora B activity was required for maintenance of a stable checkpoint response to taxol, but not when microtubules were completely disassembled with agents such as nocodazole (although see [104]).

Similar defects were observed in the checkpoint response to taxol when Survivin levels were lowered by RNAi [107,108], but a more recent study has shown that the situation is slightly more complex. In DT40 cells with a conditional knockout of Survivin, the checkpoint response to microtubule depolymerisation is normal, but so is the response to high doses of taxol [71]. Defects in the checkpoint were observed only at low doses of taxol, possibly suggesting that Aurora B activity is required primarily to amplify weak checkpoint signals, for example when the occasional bi-oriented kinetochore briefly releases its microtubules due to defective tension. DT40 cells have only four microtubules per kinetochore [109], and they may therefore represent a sensitised system where detachment occurs more readily and Aurora B activity is less important to stimulate the formation of completely unattached kinetochores.

### Sister chromatid and centromeric cohesion

Once chromosome bi-orientation is complete and the SAC is satisfied, cells start the destruction of cyclins and other APC/C substrates, and culminating in the onset of anaphase with the release of sister chromatid cohesion. Several studies have shown that Aurora B participates in the control of sister chromatid cohesion [110,111]. In vertebrates, this cohesion is released during two different stages of mitosis [112]. In prophase, Aurora B together with Plk1 controls the dissolution of cohesion between chromosome arms [110,113]. Centromeric cohesion is maintained/protected until anaphase onset and requires the presence of the Shugoshin protein SGO1 whose localisation depends on PP2A phosphatase, Aurora B and BUB1 [114–117].

The release of sister chromatid cohesion is triggered by separase after the degradation of securin through the APC/C (anaphase promoting complex/cyclosome). Recently Aurora B was shown to be involved in regulating the association of separase with mitotic chromosomes [118].

### Cleavage furrow ingression and cytokinesis

During anaphase, Aurora B concentrates at the spindle midzone and equatorial cortex, accumulating ultimately at the midbody. This localisation of the protein is essential for late mitotic events. Indeed, Aurora B and all of the CPC components have essential roles in cytokinesis in a wide range of organisms [5,6,56,119,120]. In anaphase, the position of the cleavage furrow is dictated by the location of a microtubule-dependent zone of local RhoA activity within the spindle midzone [121,122]. Using a FRET-based reporter for Aurora B activity, an anaphase Aurora B gradient has been observed on the spindle midzone. Aurora B activity across this gradient is maintained through interaction with midzone microtubules and signals the positioning of the cleavage furrow [85^••^]. The chain of events leading to the activation of RhoA and therefore to the contraction of the acto-myosin ring is regulated by Aurora B through phosphorylation of the centralspindlin complex composed of the kinesin MKLP1/ZEN4 and the Rac GTPase activating protein 1 (MgcRacGAP) [123,124].

Aurora B is also a key regulator of abscission timing if unsegregated chromatin is trapped at the furrow ingression site in human cells. In cells with chromatin bridges between daughter nuclei, active Aurora B persists in the intercellular bridge together with phosphorylated MKLP1 [125^•^]. How this stabilises the bridge is not known, but small molecule inhibition of Aurora B advances the time of abscission in otherwise unperturbed mitosis. The persistence of the Aurora B and stabilisation of the bridge depends on chromatin, as severing the chromatin of the bridge with a laser leads to subsequent abscission.

Similarly, in *S. cerevisiae*, Aurora/Ipl1 is required for the NoCut checkpoint, which can detect either spindle damage leading to failures in chromosome segregation or other problems (e.g. topoisomerase II mutants or uncleavable cohesin) that prevent the complete segregation of the sister chromatids [126,127^•^]. Ipl1 activity on the central spindle is required for the location of the proteins Boi1 and Boi2 to the bud neck, where they inhibit abscission.

## Conclusions

Since first discovered more than a decade ago [128,21] the Aurora kinase family has emerged as a major controller of the cell cycle and mitosis. As discussed here, regulation of the two major branches of the family is quite different, with Aurora B functioning within the confines of the CPC, whereas Aurora A interacts with many different partners at different times and places. These highly versatile kinases will no doubt continue to reward further detailed study for years to come.

## References and recommended reading

Papers of particular interest, published within the period of review, have been highlighted as:

• of special interest
•• of outstanding interest
